# Supplementary material for: The molecular clock of Mycobacterium tuberculosis
Source: PLoS Pathog. 2019 Sep 12;15(9):e1008067. doi: 10.1371/journal.ppat.1008067 (PMC6759198; doi:10.1371/journal.ppat.1008067)
Supplement: S3 Table — (DOCX) [file ppat.1008067.s003.docx]

| Lineage | LSD^1^ | BEAST^2^ | LSD + aDNA^3^ | BEAST + aDNA^4^ |
| --- | --- | --- | --- | --- |
| MTBC | -2287  [-3742, -1849] | NA | -1449  [-2414, -812] | NA |
| L1 | 1245  [510, 1470] | 1178  [-113, 1827] | 327 | -230  [-1354, 489] |
| L2 | -358  [-9035, 587] | 1133  [179, 1559] | 575 | 114  [-809, 732] |
| L3 | 1006  [-949, 1385] | -632  [-4665, 683] | 983 | 638  [-55, 1111] |
| L4 | -410  [-1906, 91] | 428  [-192, 860] | 582 | 200  [-676, 779] |
| L5 | NA | NA | 1131 | 877  [29, 1250] |
| L6 | NA | NA | 633 | 181  [-766, 809] |
| *M. bovis* | 692  [297, 849] | 959  [344, 1431] | 551 | 936  [374, 1286] |

^1^ Age of the most recent common ancestor (negative values = BC, positive values = AD), point estimate and 95% CI. These estimates refer to the individual analyses performed on each data set.

^2^ Age of the most recent common ancestor (negative values = BC, positive values = AD), median value and 95% HPD. These estimates refer to the individual analyses performed on each data set. Since BEAST placed the root in the “wrong” position we have no estimates for the MTB complex. The results for L3 refer to the analysis with constant population size, all other data sets rejected the constant population size model, therefore we report the results of the exponential population growth analysis.

^3^ Age of the most recent common ancestor (negative values = BC, positive values = AD), point estimate and 95% CI. These estimates refer to a single analysis performed on the complete data set of 6,285 strains + 3 aDNA samples; LSD outputs confidence intervals only for the MRCA of the tree and not for the nodes.

^4^ Age of the most recent common ancestor (negative values = BC, positive values = AD), median value and 95% HPD. These estimates refer to a single analysis performed on the random subset of MTBC composed of 500 strains + 3 aDNA samples; since BEAST placed the root in the “wrong” position we have no estimates for the MTB complex. Nevertheless we could retrieve the age of the MRCA of the individual lineages.
