# Supplementary figures and images for: The molecular clock of Mycobacterium tuberculosis
Source: PLoS Pathog. 2019 Sep 12;15(9):e1008067. doi: 10.1371/journal.ppat.1008067 (PMC6759198; doi:10.1371/journal.ppat.1008067)

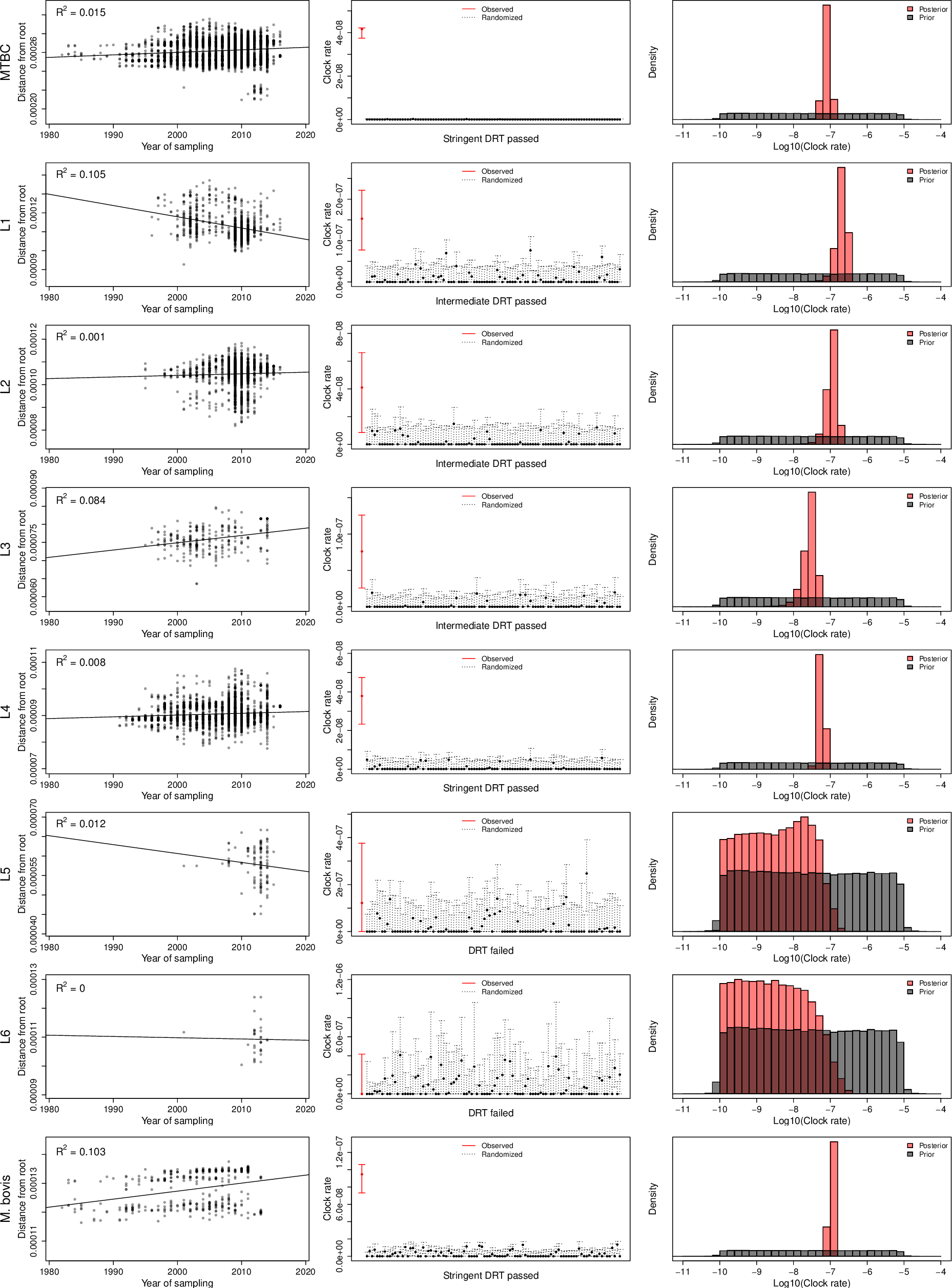

Supplement: S1 Fig — The simple DRT is passed when the clock rate estimate for the observed data does not overlap with the range of estimates obtained from the randomized sets. The intermediate DRT is passed when the clock rate estimate for the observed data does not overlap with the confidence intervals of the estimates obtained from the randomized sets. The stringent DRT is passed when the confidence interval of the clock rate estimate for the observed data does not overlap with the confidence intervals of the estimates obtained from the randomized sets. Large data sets (MTBC, L1, L2, L4 and M. bovis) were randomly sub-sampled to 300 strains for the BEAST analysis. (TIF) [file ppat.1008067.s005.tif]

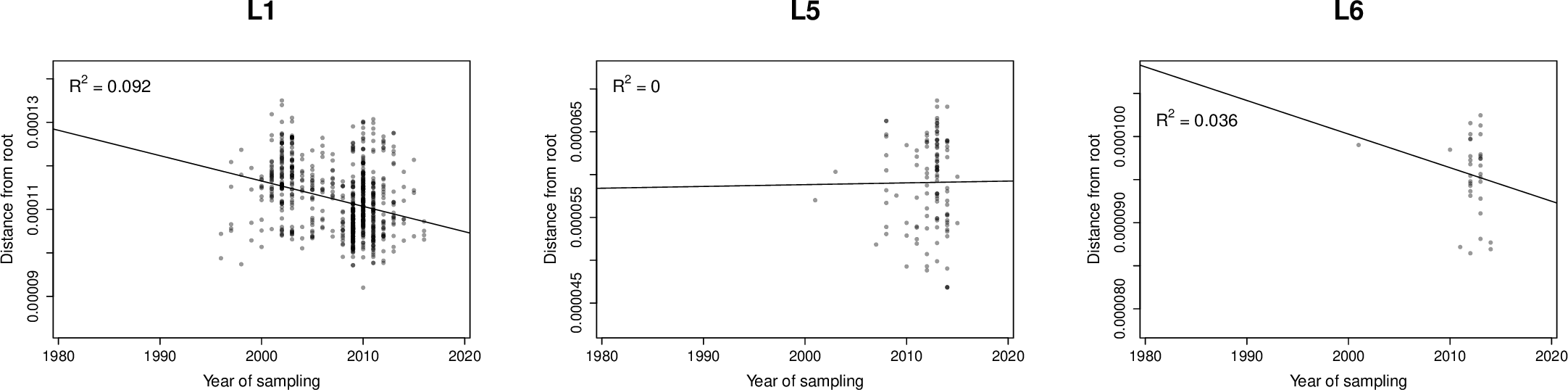

Supplement: S2 Fig — The difference compared to S1 Fig is that the root was not placed in the position that minimizes the sum of the squared residuals from the regression line, but was obtained from the complete MTBC tree as shown in Fig 3a, and it is therefore defined by the outgroup of each of these lineages. (TIF) [file ppat.1008067.s006.tif]

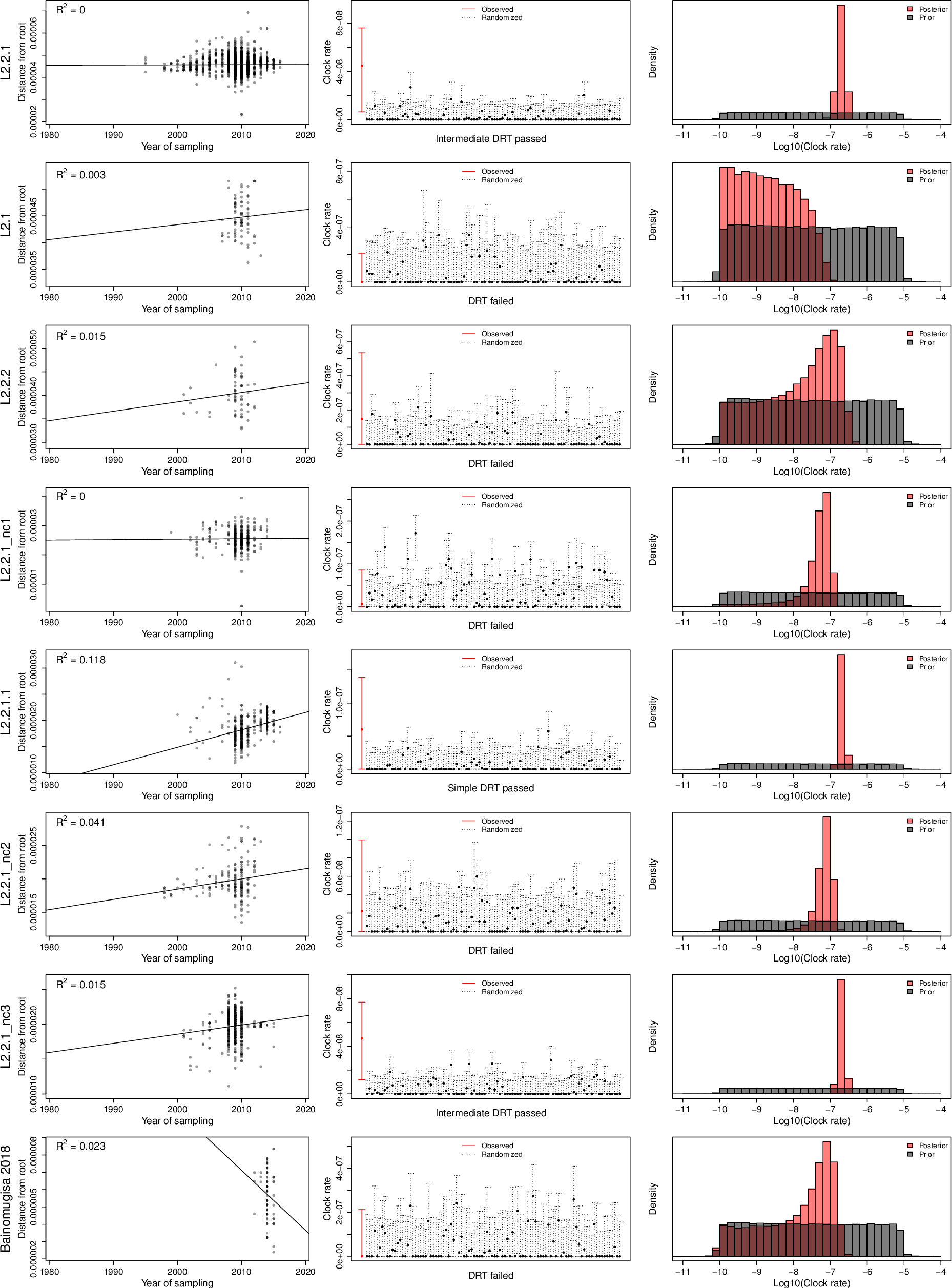

Supplement: S3 Fig — The simple DRT is passed when the clock rate estimate for the observed data does not overlap with the range of estimates obtained from the randomized sets. The intermediate DRT is passed when the clock rate estimate for the observed data does not overlap with the confidence intervals of the estimates obtained from the randomized sets. The stringent DRT is passed when the confidence interval of the clock rate estimate for the observed data does not overlap with the confidence intervals of the estimates obtained from the randomized sets. Large data sets (L1.1.1, L1.1.1.1, L2.2.1, L2.2.1.1, L2.2.1_nc1, L2.2.1_nc3, L4.10, L4.1.2) were randomly sub-sampled to 300 strains for the BEAST analysis. (TIF) [file ppat.1008067.s007.tif]

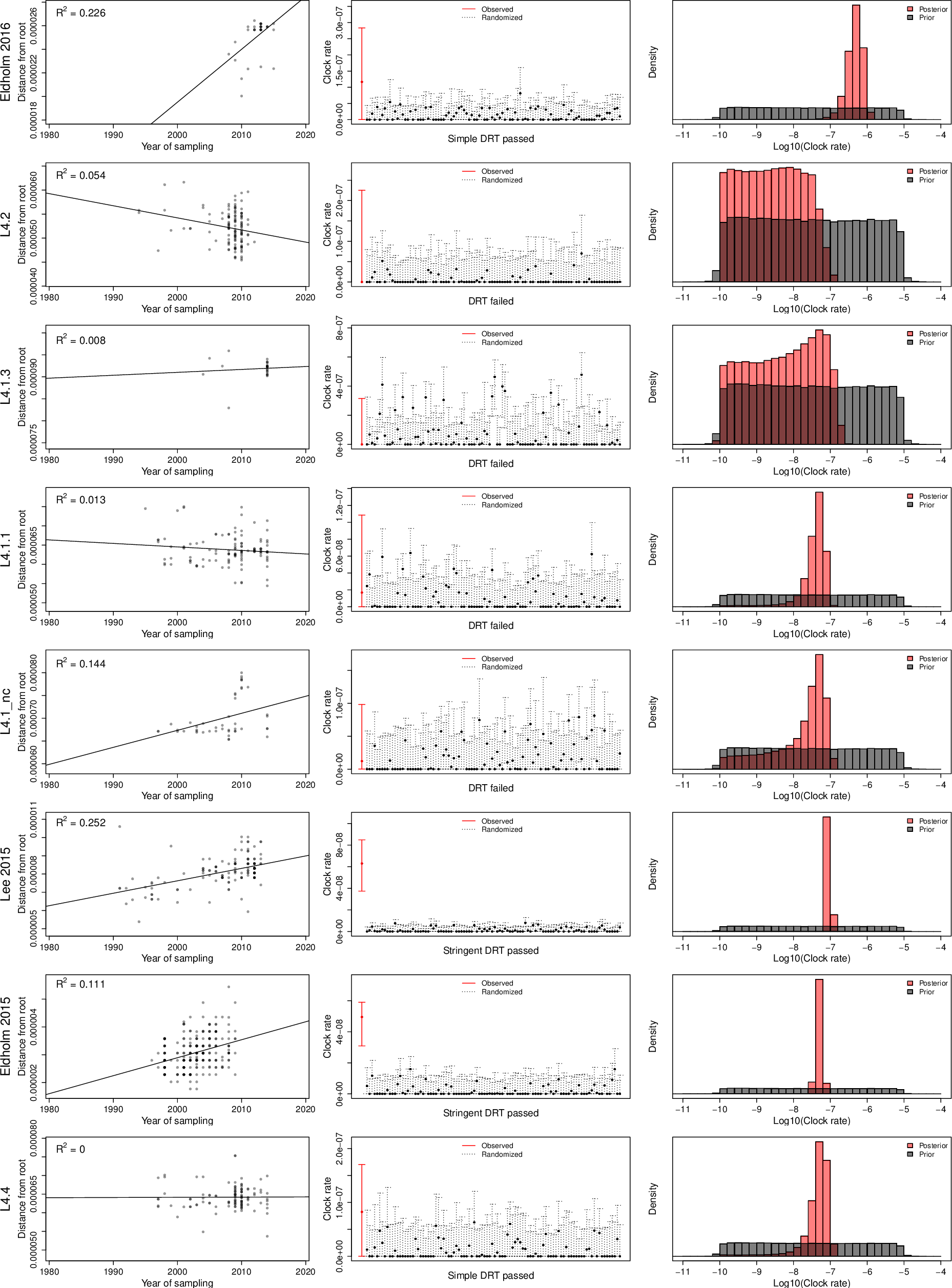

Supplement: S4 Fig — The simple DRT is passed when the clock rate estimate for the observed data does not overlap with the range of estimates obtained from the randomized sets. The intermediate DRT is passed when the clock rate estimate for the observed data does not overlap with the confidence intervals of the estimates obtained from the randomized sets. The stringent DRT is passed when the confidence interval of the clock rate estimate for the observed data does not overlap with the confidence intervals of the estimates obtained from the randomized sets. Large data sets (L1.1.1, L1.1.1.1, L2.2.1, L2.2.1.1, L2.2.1_nc1, L2.2.1_nc3, L4.10, L4.1.2) were randomly sub-sampled to 300 strains for the BEAST analysis. (TIF) [file ppat.1008067.s008.tif]

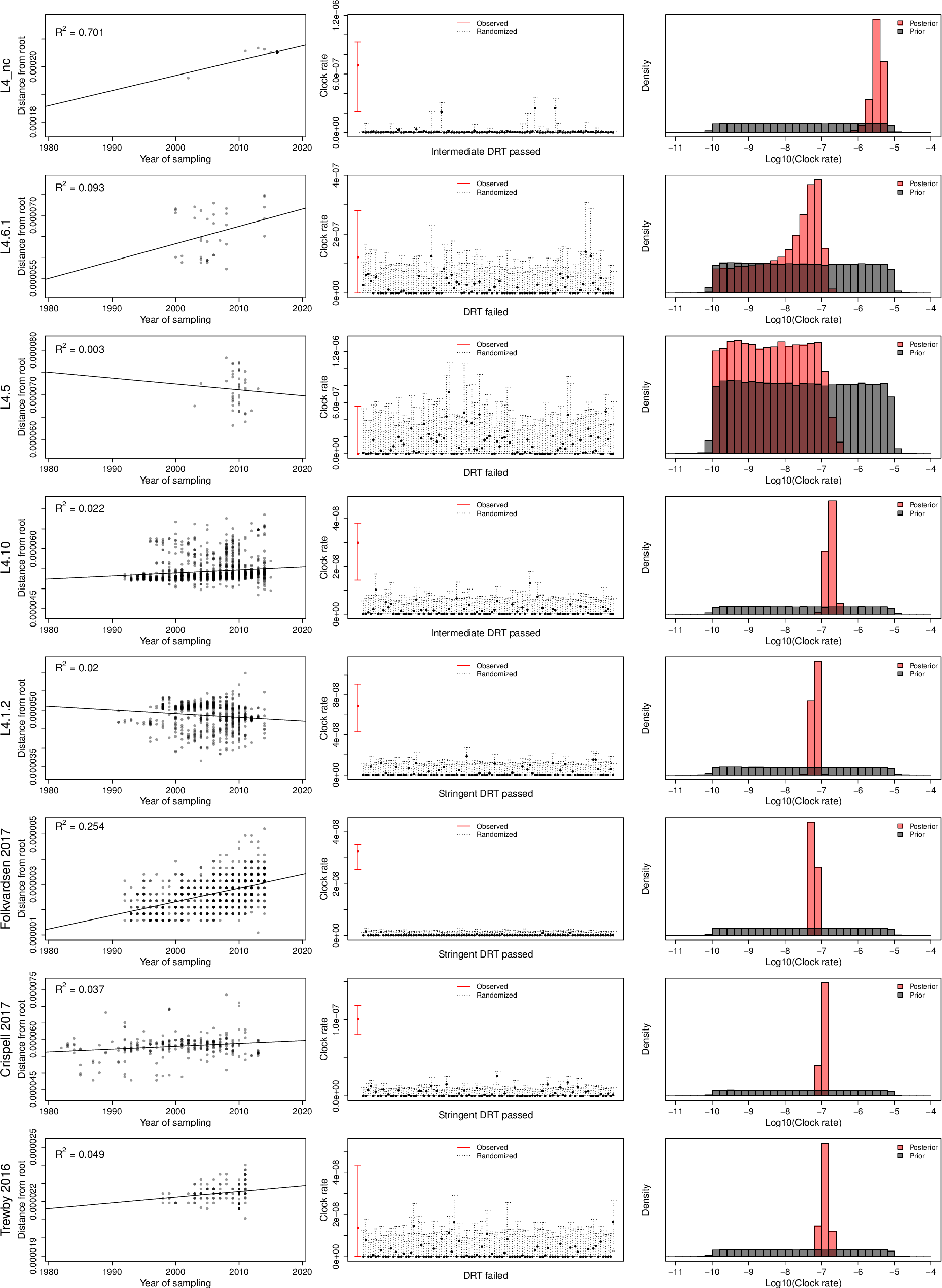

Supplement: S5 Fig — The simple DRT is passed when the clock rate estimate for the observed data does not overlap with the range of estimates obtained from the randomized sets. The intermediate DRT is passed when the clock rate estimate for the observed data does not overlap with the confidence intervals of the estimates obtained from the randomized sets. The stringent DRT is passed when the confidence interval of the clock rate estimate for the observed data does not overlap with the confidence intervals of the estimates obtained from the randomized sets. Large data sets (L1.1.1, L1.1.1.1, L2.2.1, L2.2.1.1, L2.2.1_nc1, L2.2.1_nc3, L4.10, L4.1.2) were randomly sub-sampled to 300 strains for the BEAST analysis. (TIF) [file ppat.1008067.s009.tif]

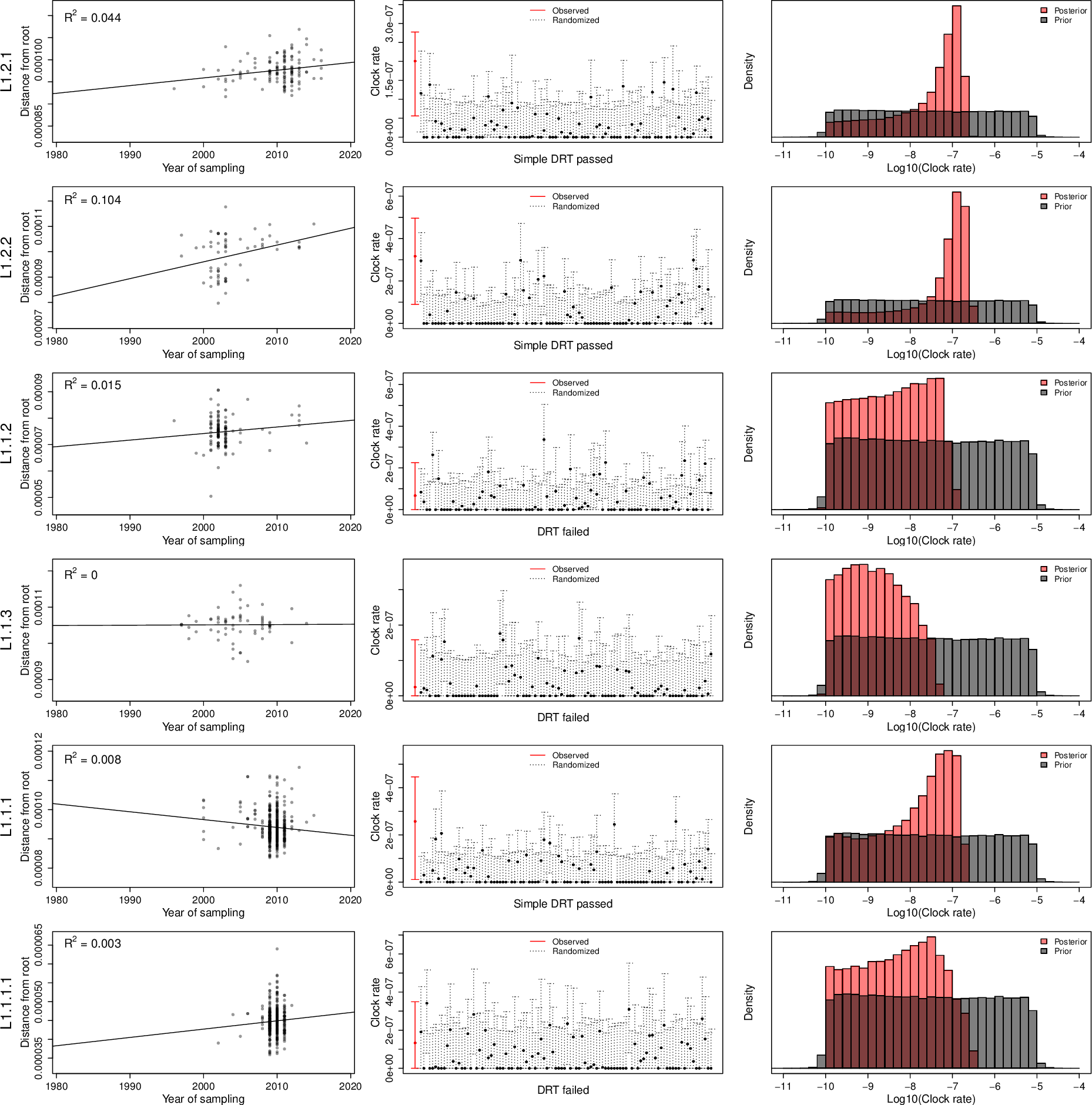

Supplement: S6 Fig — The simple DRT is passed when the clock rate estimate for the observed data does not overlap with the range of estimates obtained from the randomized sets. The intermediate DRT is passed when the clock rate estimate for the observed data does not overlap with the confidence intervals of the estimates obtained from the randomized sets. The stringent DRT is passed when the confidence interval of the clock rate estimate for the observed data does not overlap with the confidence intervals of the estimates obtained from the randomized sets. Large data sets (L1.1.1, L1.1.1.1, L2.2.1, L2.2.1.1, L2.2.1_nc1, L2.2.1_nc3, L4.10, L4.1.2) were randomly sub-sampled to 300 strains for the BEAST analysis. (TIF) [file ppat.1008067.s010.tif]

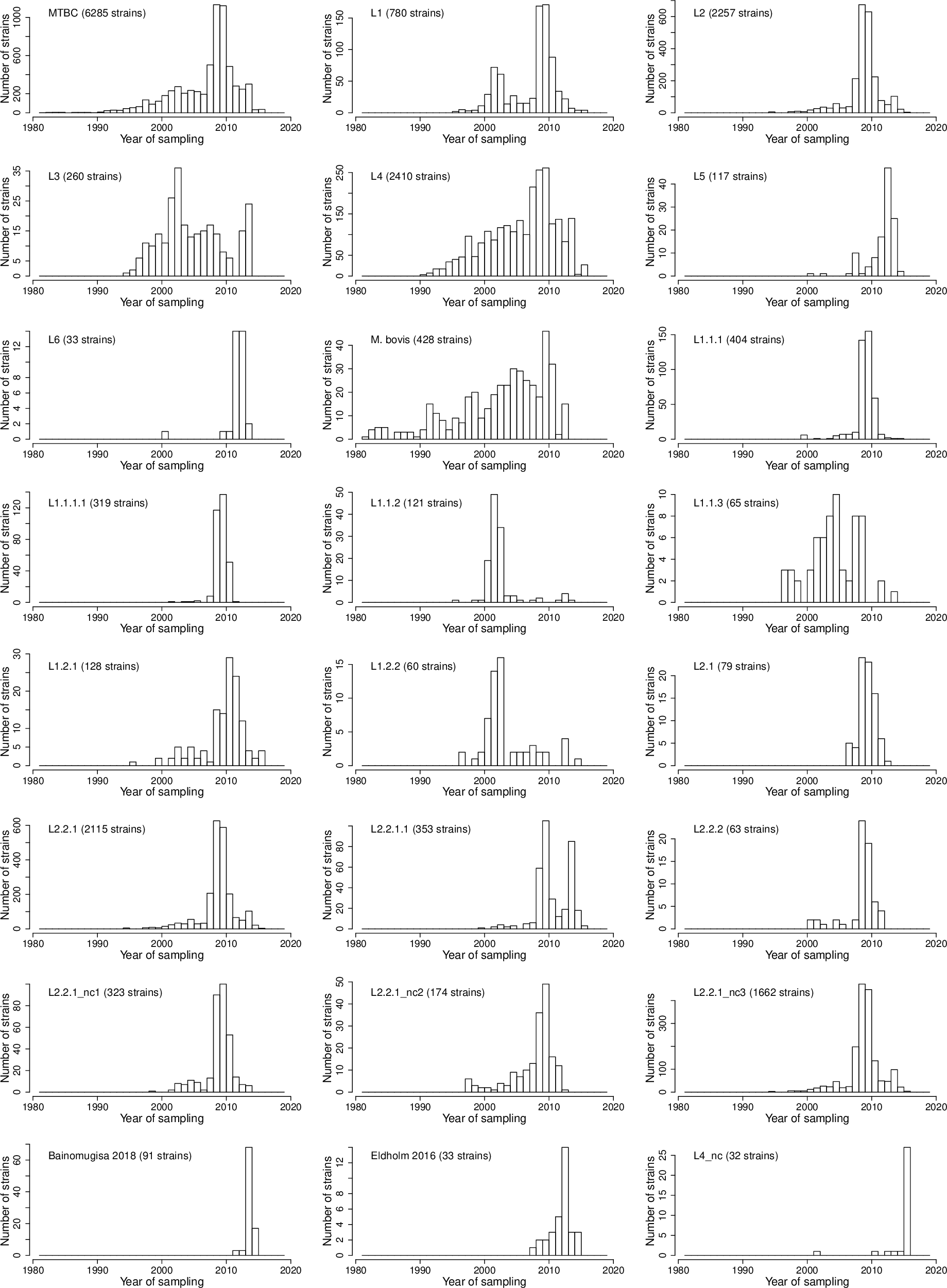

Supplement: S7 Fig — (TIF) [file ppat.1008067.s011.tif]

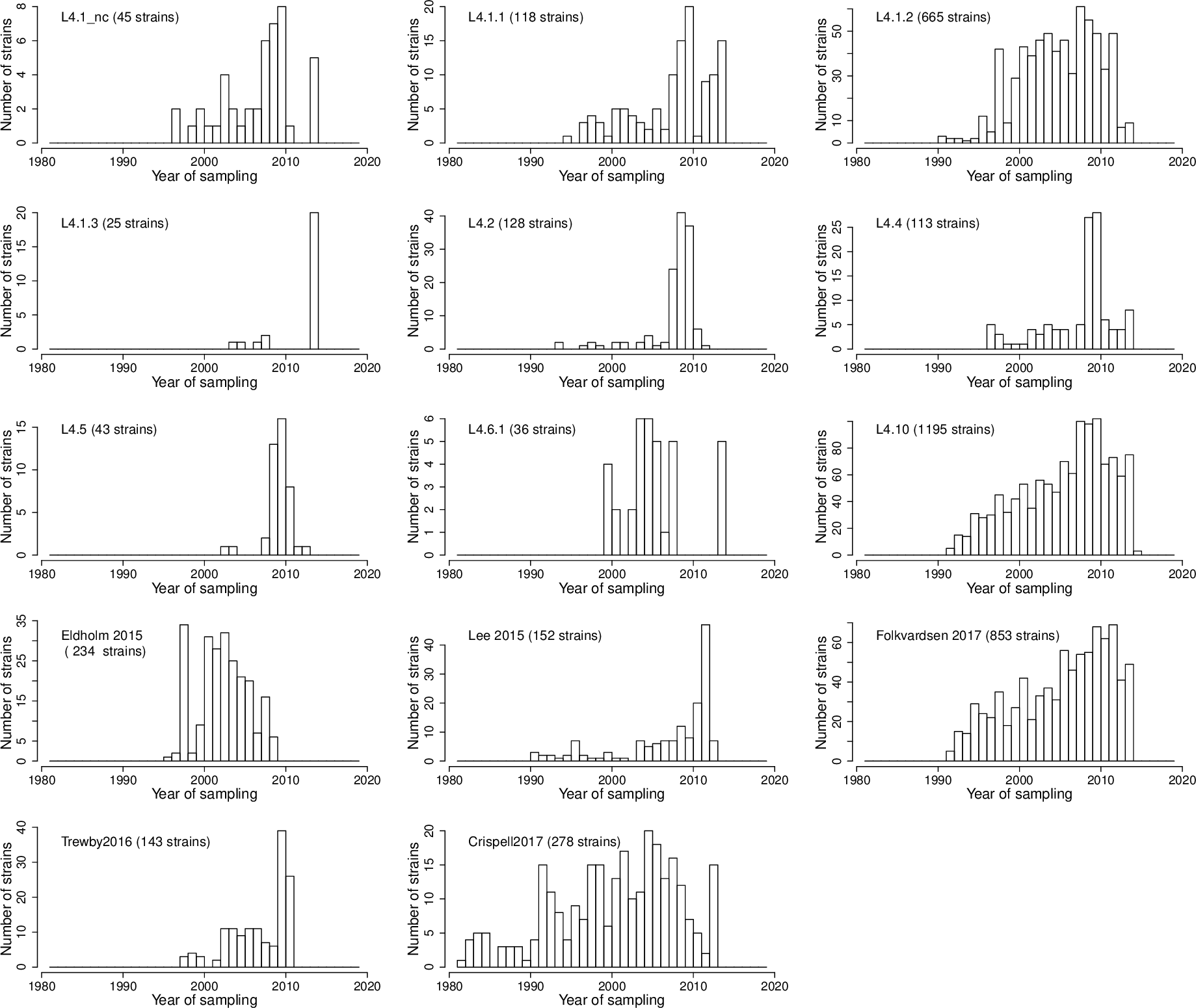

Supplement: S8 Fig — (TIF) [file ppat.1008067.s012.tif]

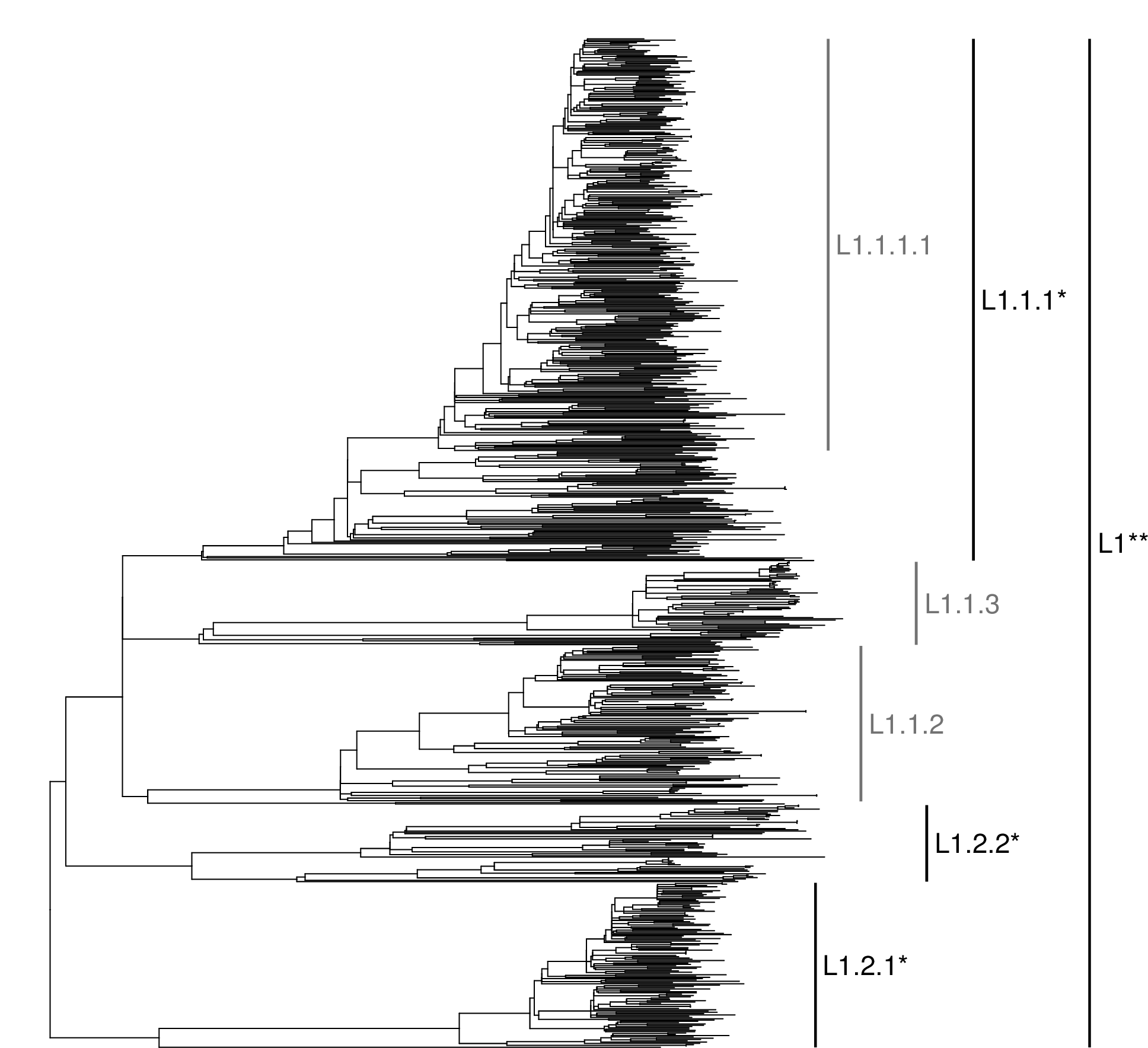

Supplement: S9 Fig — Clades colored in gray did not pass the DRT, clades colored in black passed the DRT. *: simple DRT passed, ** intermediate DRT passed, ***: stringent DRT passed. (TIF) [file ppat.1008067.s013.tif]

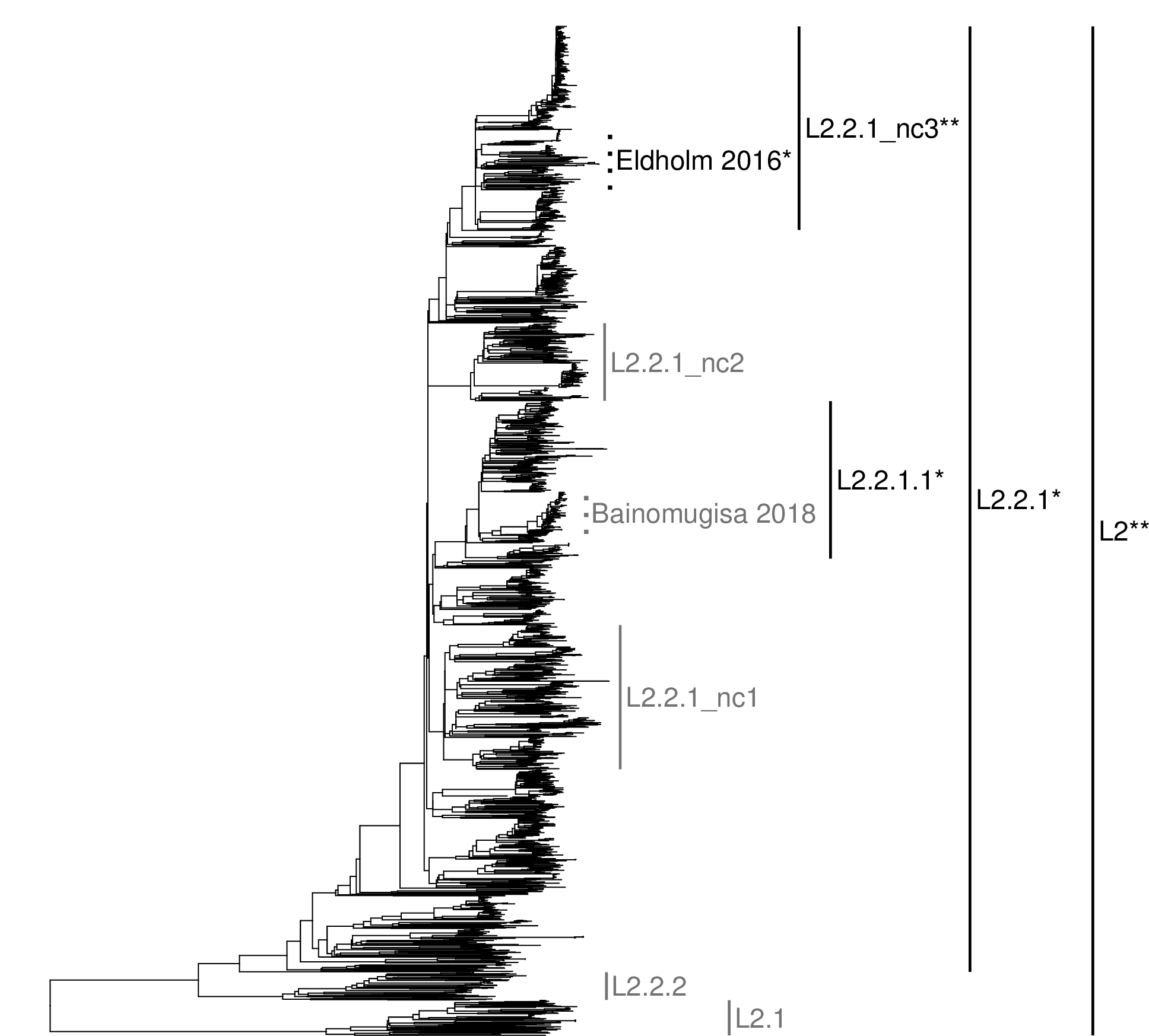

Supplement: S10 Fig — Clades colored in gray did not pass the DRT, clades colored in black passed the DRT. *: simple DRT passed, ** intermediate DRT passed, ***: stringent DRT passed. Dotted lines represent two outbreaks from previous studies. (TIF) [file ppat.1008067.s014.tif]

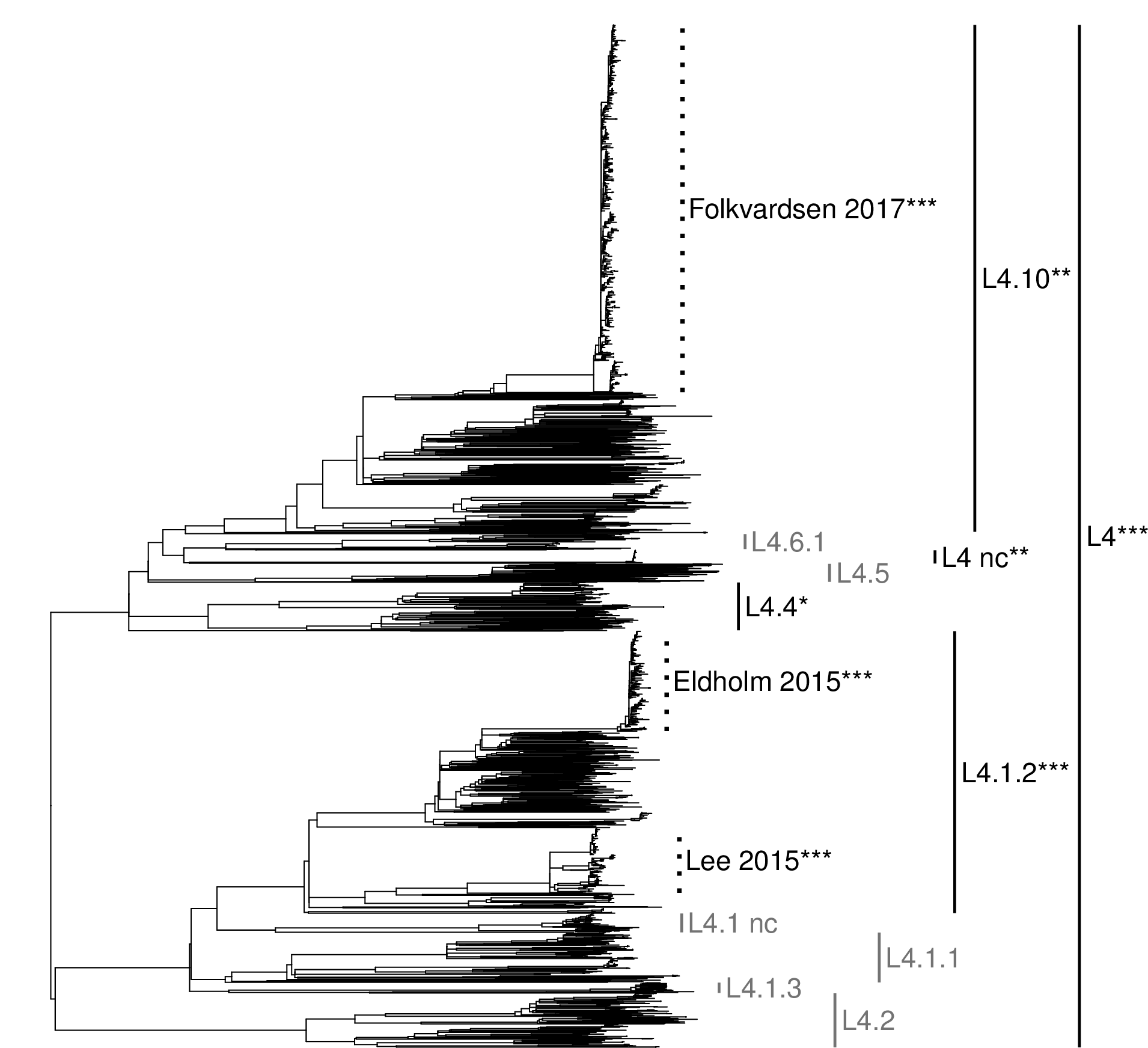

Supplement: S11 Fig — Clades colored in gray did not pass the DRT, clades colored in black passed the DRT. *: simple DRT passed, ** intermediate DRT passed, ***: stringent DRT passed. Dotted lines represent three outbreaks from previous studies. (TIF) [file ppat.1008067.s015.tif]

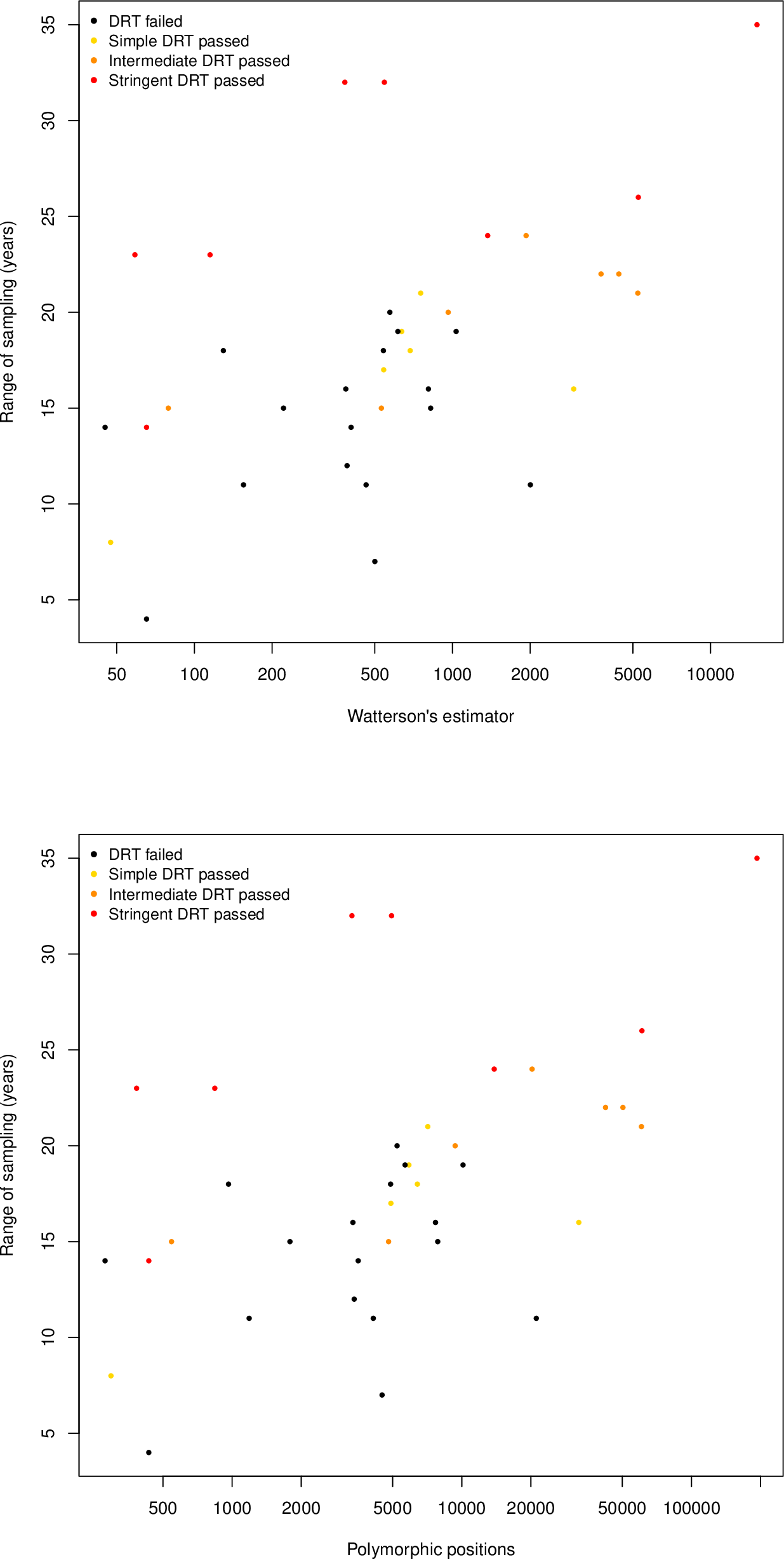

Supplement: S12 Fig — Data sets with fewer strains sampled in a shorter period of time tended to fail the DRT irrespectively of the genetic diversity of the data set. (TIF) [file ppat.1008067.s016.tif]

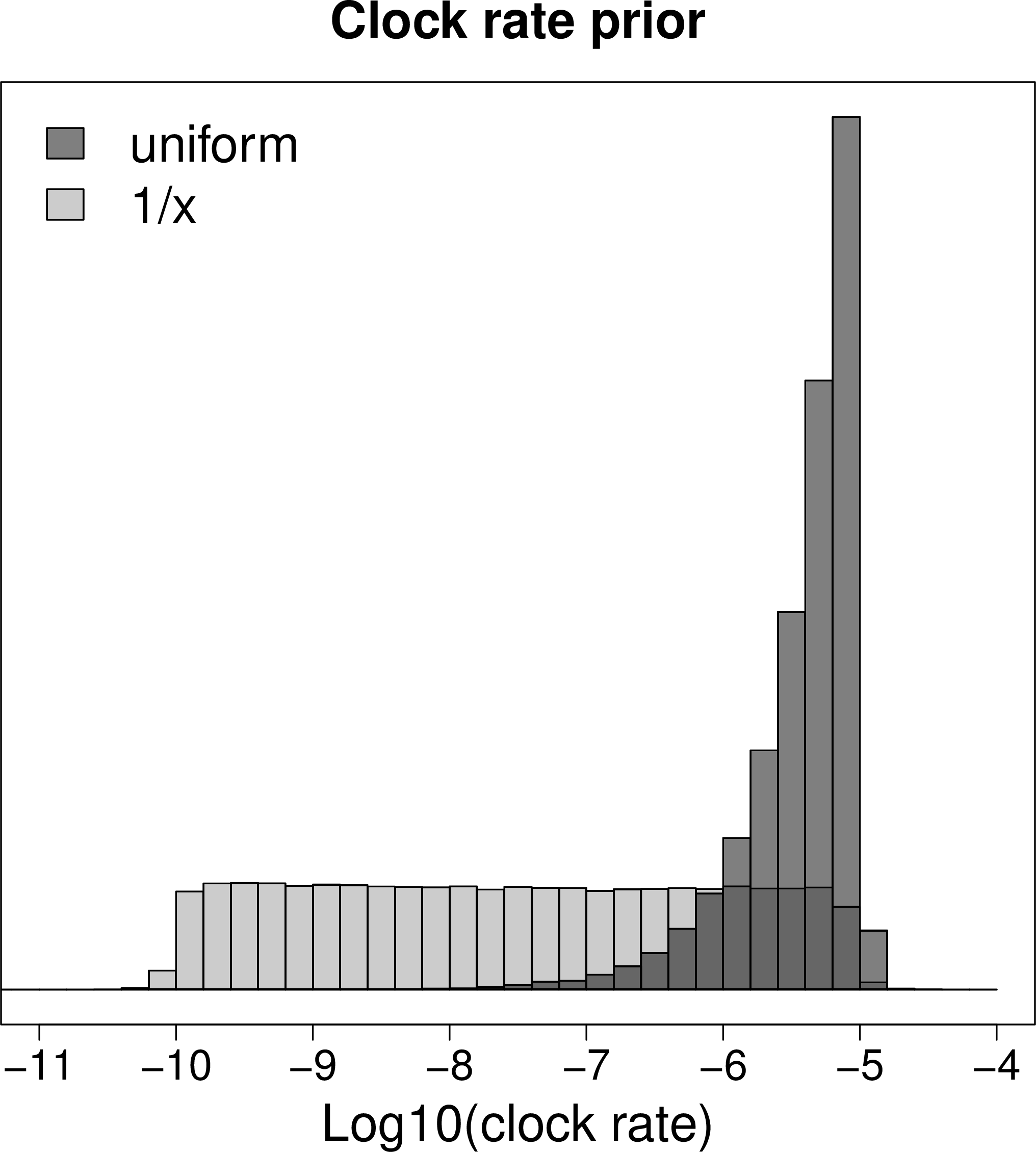

Supplement: S13 Fig — The uniform prior place most weight on high clock rates, while the 1/x prior distributes the weight through all orders of magnitude. (TIF) [file ppat.1008067.s017.tif]

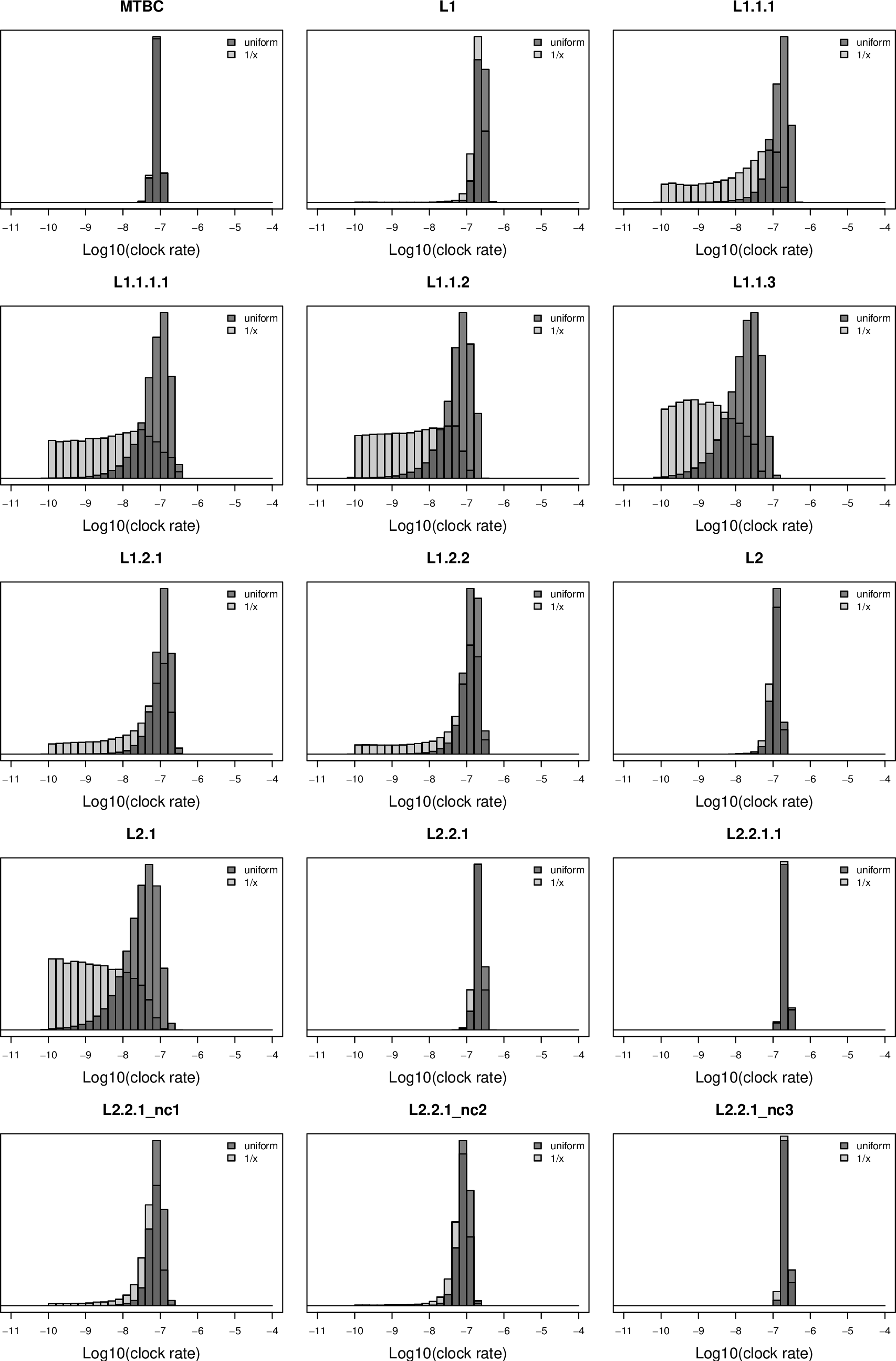

Supplement: S14 Fig — The prior distributions for the two analyses are shown in S13 Fig. (TIF) [file ppat.1008067.s018.tif]

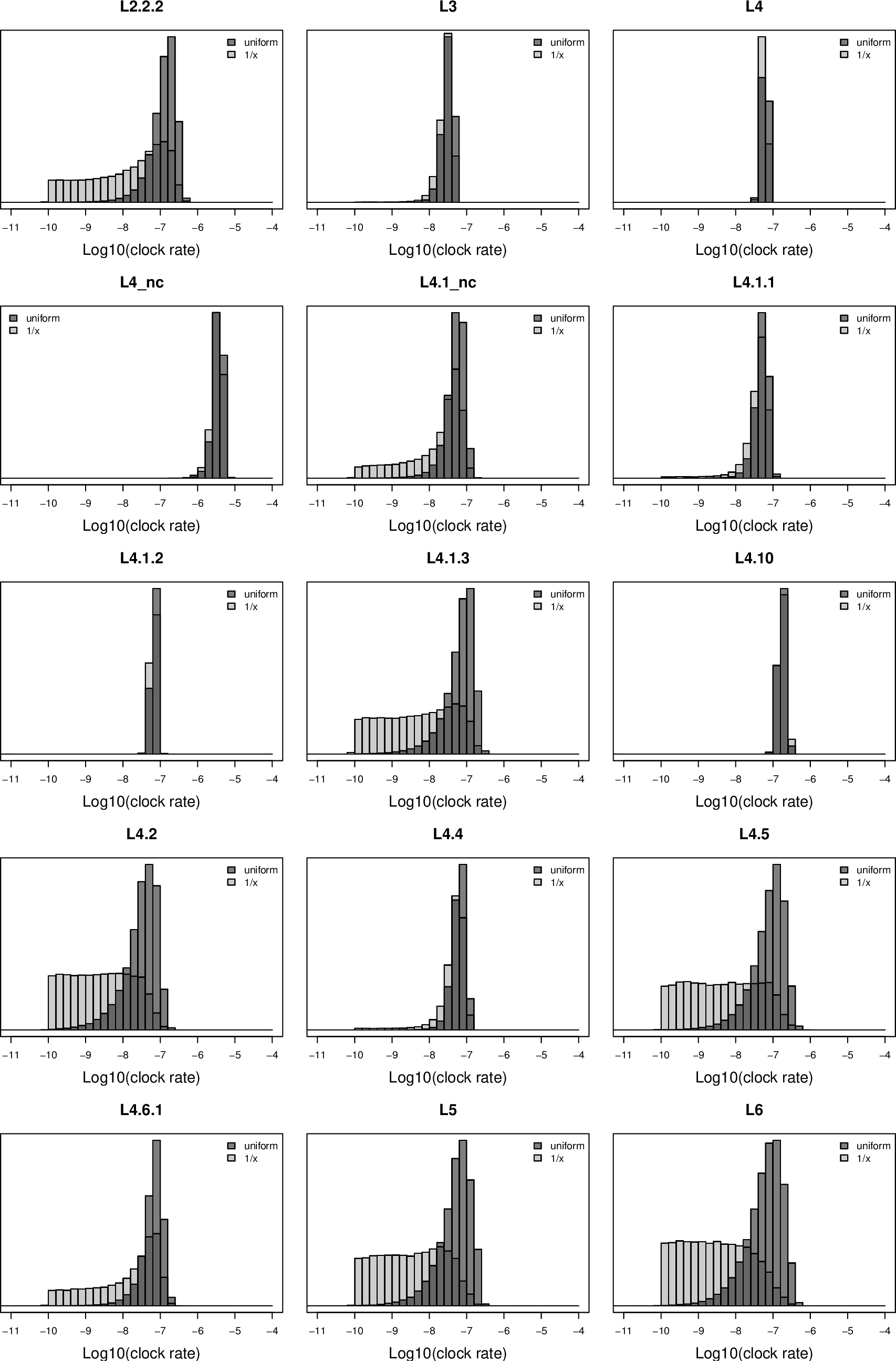

Supplement: S15 Fig — The prior distributions for the two analyses are shown in S13 Fig. (TIF) [file ppat.1008067.s019.tif]

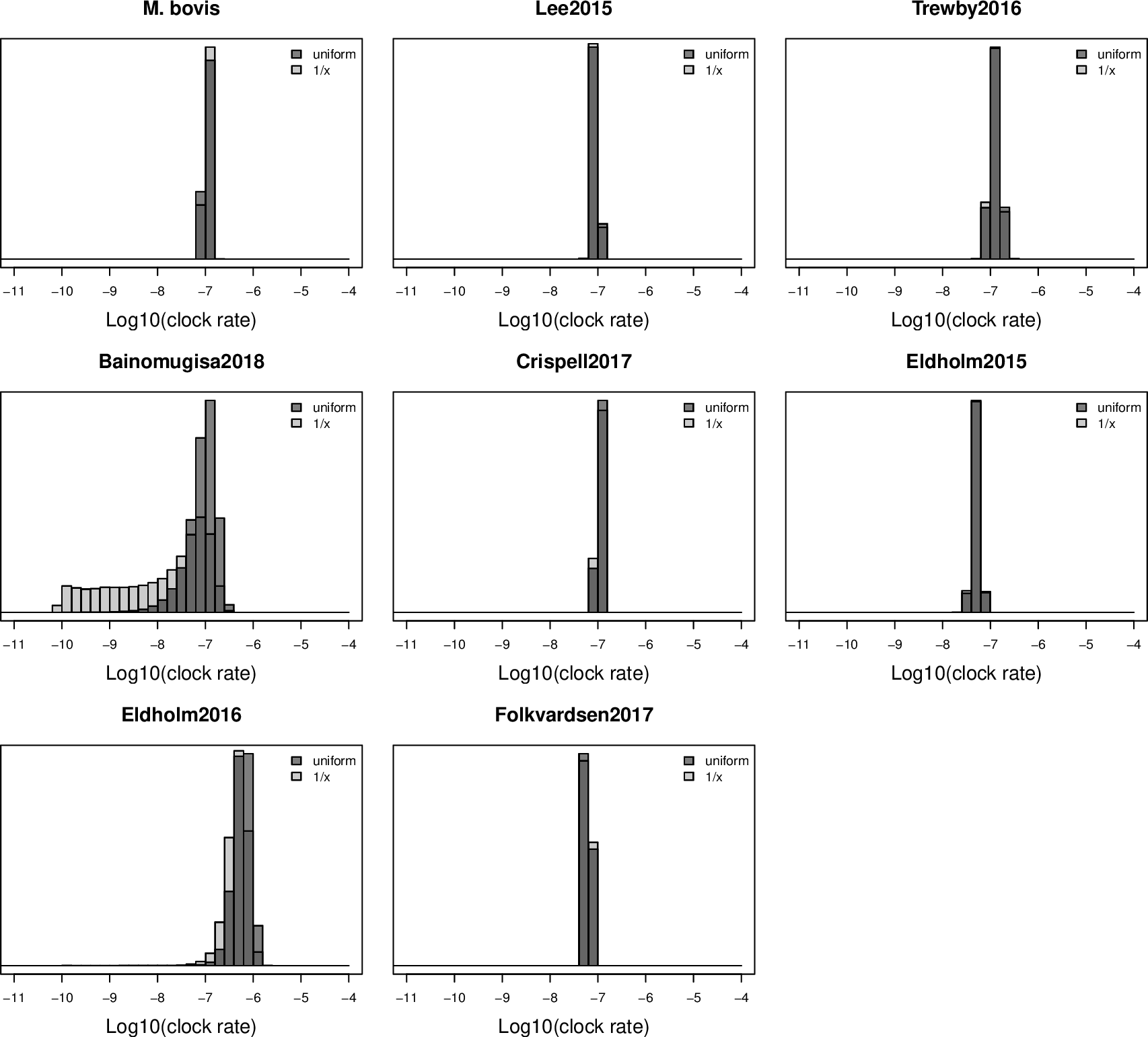

Supplement: S16 Fig — The prior distributions for the two analyses are shown in S13 Fig. (TIF) [file ppat.1008067.s020.tif]

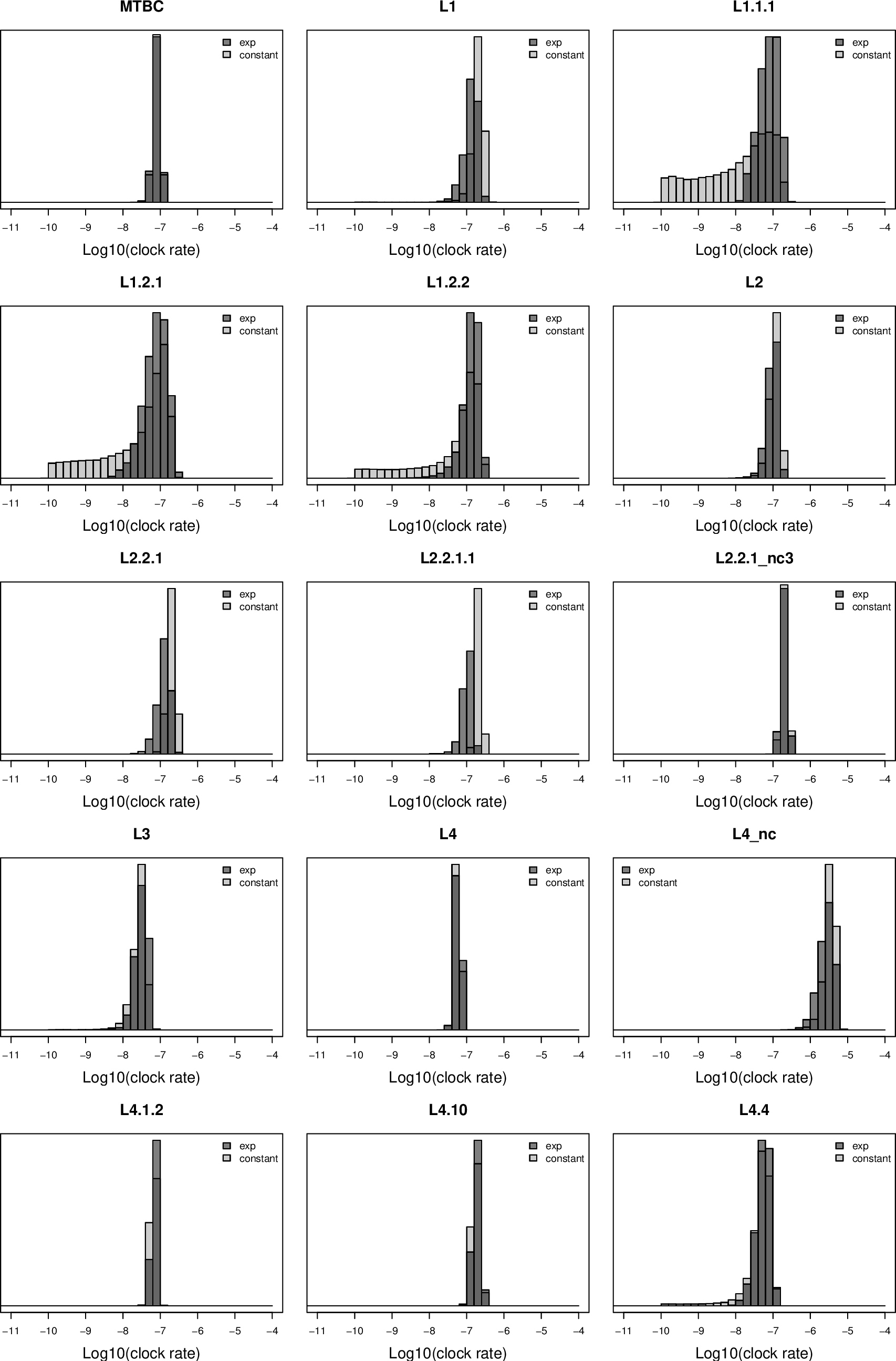

Supplement: S17 Fig — (TIF) [file ppat.1008067.s021.tif]

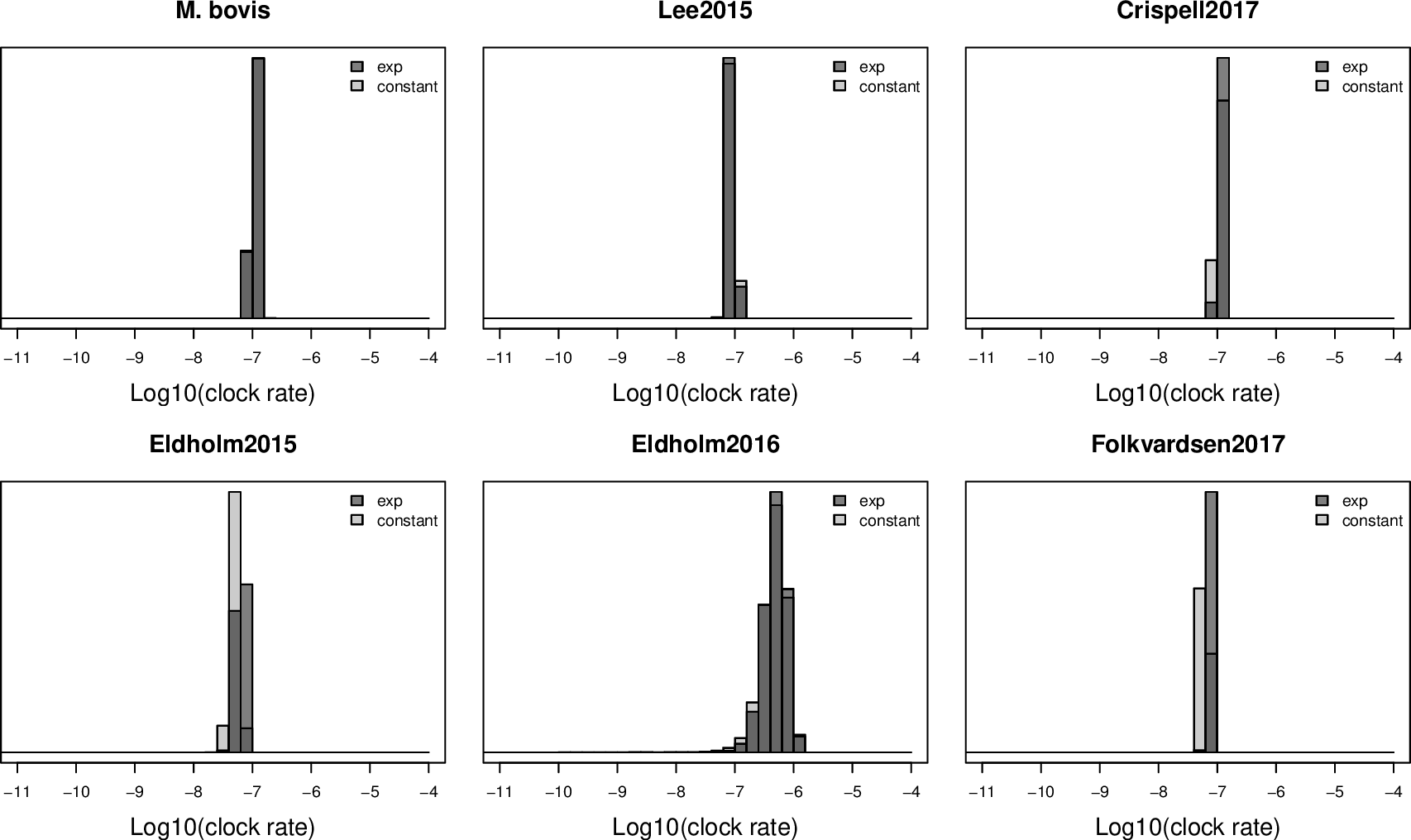

Supplement: S18 Fig — (TIF) [file ppat.1008067.s022.tif]

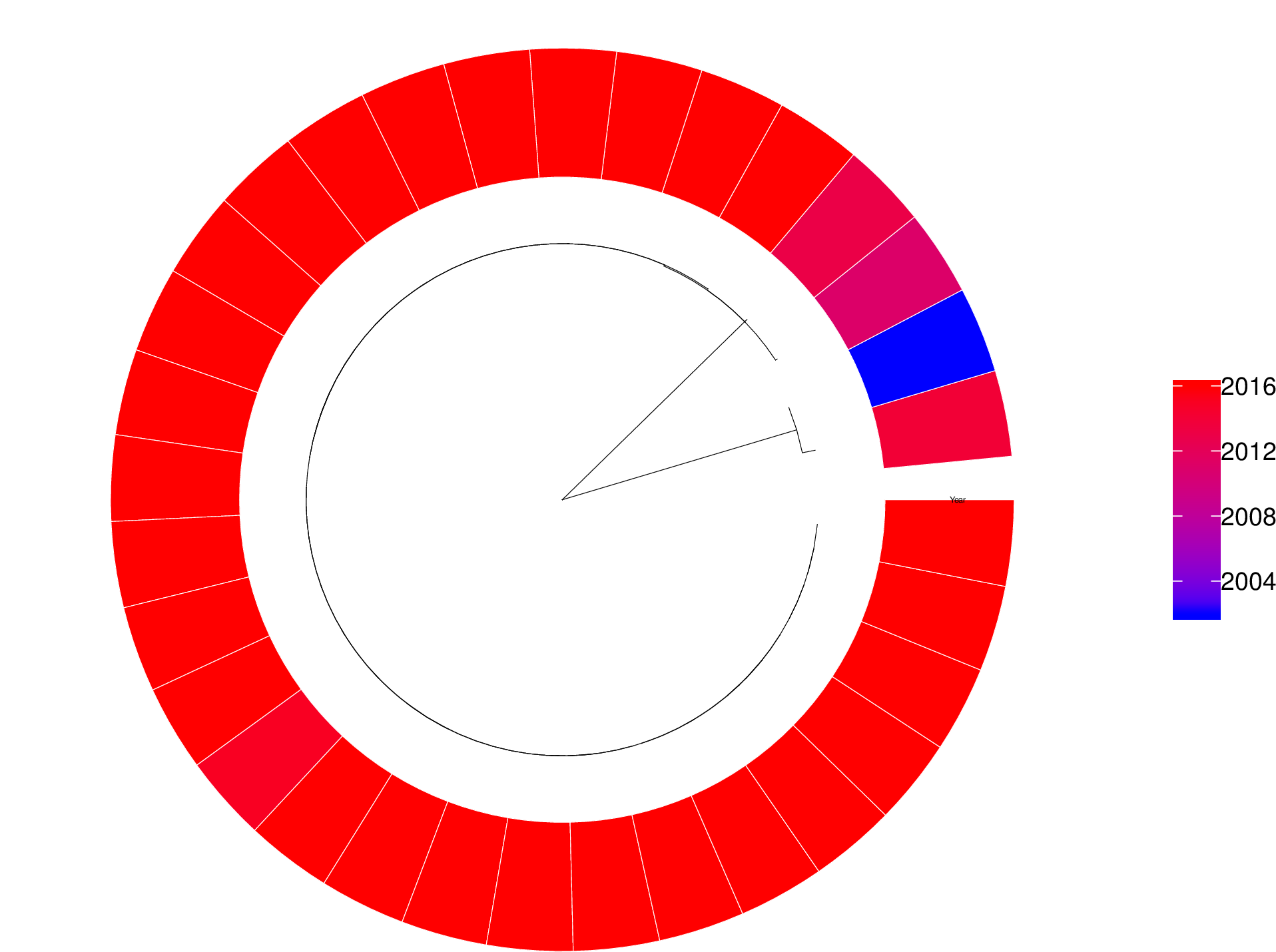

Supplement: S19 Fig — (TIF) [file ppat.1008067.s023.tif]

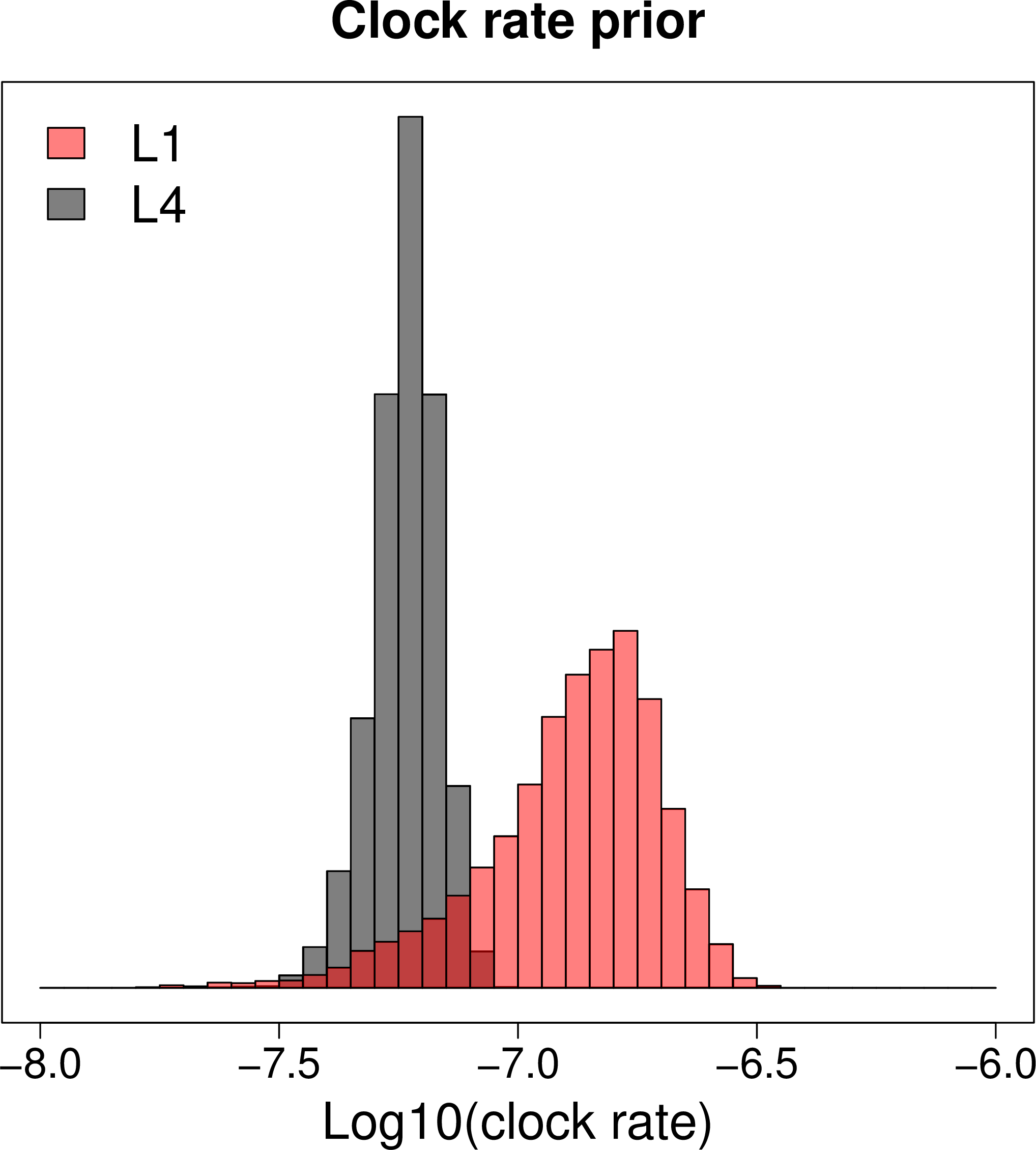

Supplement: S20 Fig — These are the results of the analysis with the 1/x prior on the clock rate and the exponential population growth (or shrinkage) prior. (TIF) [file ppat.1008067.s024.tif]
